# The Changes of Liver Stiffness and Its Associated Factors for Chronic Hepatitis B Patients with Entecavir Therapy

**DOI:** 10.1371/journal.pone.0093160

**Published:** 2014-03-28

**Authors:** Yuan-Hung Kuo, Sheng-Nan Lu, Chien-Hung Chen, Kuo-Chin Chang, Chao-Hung Hung, Wei-Chen Tai, Ming-Chao Tsai, Po-Lin Tseng, Tsung-Hui Hu, Jing-Houng Wang

**Affiliations:** Division of Hepato-Gastroenterology, Department of Internal Medicine, Kaohsiung Chang Gung Memorial Hospital and Chang Gung University College of Medicine, Kaohsiung City, Taiwan; Kaohsiung Medical University Hospital, Kaohsiung Medical University, Taiwan

## Abstract

Liver stiffness measurement (LSM) using transient elastography has been proposed to assess liver fibrosis well in various liver diseases. This study was to determine the changes of LSM and its associated factors for chronic hepatitis B (CHB) patients undergoing Entecavir therapy. Consecutive CHB patients underwent Entecavir therapy with two LSMs were enrolled. Patients with aspartate transaminase (AST) and/or alanine transaminase ≧200 IU/L were excluded. The retrospective study enrolled 233 patients including 132 without cirrhosis (group 1) and 101 with cirrhosis (group 2). The mean values of initial liver stiffness were 7.9 and 16.6 kPa for patients in group 1 and group 2, respectively (p<0.001). In addition to the decline of transaminase levels, there was significant reduction of liver stiffness value in a mean interval of 52.8 and 61.9 weeks between the two LSMs for patients in group 1 and 2, respectively (p<0.001). Multivariate analysis showed that higher initial LSM value and presence of hepatitis B e-antigen were associated with a greater decline of LSM value, whereas follow-up AST≧40 IU/L with increased LSM value for group 1 patients. For group 2 patients, longer interval between the two LSMs, higher initial LSM value and AST≧40 IU/L were associated with a greater decline of LSM value, whereas presence of diabetes mellitus (DM) contributed to increased LSM value. In conclusion, CHB patients improved their LSM values after Entecavir therapy. Higher initial LSM value contributed to greater LSM reduction. However, in cirrhotic patients, DM was associated with an increased LSM value after therapy.

## Introduction

Chronic infection with hepatitis B virus (HBV) currently affects about 400 million people worldwide, and leads to complications of cirrhosis, hepatic decompensation and hepatocellular carcinoma (HCC).[1], [2] The severity of liver fibrosis is associated with the prognosis of liver disease. HBV-related HCC develops in more than 60% of patients with cirrhosis, so-called advanced fibrosis.[3] Long-term suppression of HBV with antiviral therapy has been shown to significantly improve the stages of fibrosis in patients with chronic HBV (CHB) infection. [4]–[6] Hence, monitoring the stage of liver fibrosis in CHB patients undergoing anti-viral therapy is important to evaluate the effectiveness of the therapy and to predict the prognosis. Liver biopsy is currently considered as the gold standard in staging fibrosis. However, liver biopsy might occasionally be associated with complication or sampling errors [7]–[9].

Liver stiffness measurement (LSM) using transient elastography has been validated as an accurate tool in assessing significant liver fibrosis and cirrhosis in various liver diseases.[10]–[14] The results of LSM were highly reproducible, with high inter-observer and intra-observer concordance.[15] In addition, serial LSMs were easy to perform and useful to follow up the patients with liver diseases.[16] Previous studies showed that the patients with chronic hepatitis C virus (HCV) infection acquiring sustained virological response (SVR) by antiviral therapy could have a significant decline of LSM values.[17], [18] Entecavir (ETV), an oral neucleotide analogue, has been proven as an effective anti-HBV drug with high potency and low resistance.[19], [20] It was also shown that the patients with CHB treated with ETV achieving an improvement in the longitudinal changes in LSM.[21]–[23] However, the factors associated with the improvement were not identified. Therefore, the present study aims to determine the changes in LSM and its associated factors for patients with CHB undergoing ETV therapy.

## Patients and Methods

### Patients

From August 2008 to February 2012, consecutive patients with CHB who underwent ETV therapy and received at least two LSMs in Kaohsiung Chang Gung Memorial hospital, Taiwan were enrolled. All patients were characterized with the presence of HBV surface antigen (HBsAg) for at least 6 months. ETV was used in a daily dose of 0.5 mg based on the treatment guideline of the Asian Pacific Association for the Study of the Liver (APASL). The patients co-infected with HCV were excluded. In addition, those with aspartate transaminase (AST) ≧200 IU/L, alanine transaminase (ALT) ≧200 IU/L, or HCC diagnosed at the time of the first LSM were excluded. The initial LSM was conducted at the enrollment, whereas the second LSM was performed in approximately one year after the initial measurement. Patients with liver cirrhosis were identified through the ultrasonography.[24] The study protocol was approved by the Institution Review Board of Kaohsiung Chang Gung Memorial hospital, and carried out in compliance with the Helsinki declaration.

### Liver stiffness measurement

LSM was conducted for patients at fasting state with a M-probe of transient elastography (FibroScan, Echosens, Paris, France). This procedure has been described previously.[25] At least 10 valid measurements were obtained for each patient. The results were included in the final analysis only if the measurement success rate was >60% in addition to the interquartile range-to-liver stiffness ratio was <0.30. The median values of the validated measurements were reported as the representative of the liver stiffness and expressed in units of kilopascals (kPa).

### Serology

The status of HBV infection such as HBsAg, quantitative HBsAg (qHBsAg), hepatitis B e-antigen (HBeAg), anti-hepatitis B e-antibody (anti-HBe Ab) and HBV DNA were detected. The presence of HBsAg was determined using commercial assay kits (HBsAg, MEIA 3.0; Abbott, North Chicago, IL, USA). qHBsAg was quantified using ARCHITECT HBsAg assay (Abbott, Chicago, IL, USA) according to manufacturer's instructions. The lower limit of detection was 0.05 IU/ml. Serum HBeAg levels were measured by a microparticle enzyme immunoassay (AxSYM; Abbott). TheAxSYM assay result was based on the ratio of the sample (S) to the cut-off (Co) (S/Co ratio) for each sample and control. The positivity of HBeAg was defined as S/Co ratio ≧ 1.0 in accordance with the manufacturer's instructions. Serum HBV DNA levels were determined using a quantitative real-time PCR assay, the COBAS AmpliPrep-COBAS TaqMan HBV test (CAP-CTM; Roche Molecular Systems Inc., Branchburg, NJ, USA), with a lower detection limit of 12 IU/mL (70 copies/mL). Dilution was performed if HBV DNA levels were >10^6^ copies/mL.

### Statistical analysis

The changes in LSM values at two points were analyzed by the Wilcoxon signed-rank test. Analyses of unpaired data were evaluated by the Mann–Whitney U-test. Data values were expressed as medians with interquartile ranges. Stepwise multiple linear regression model was used to characterize the independent factors which influenced the events. All statistical analyses were performed with SPSS software. (SPSS, version 18.0). Statistical significance was set at p<0.05.

## Results

### Patients

A total of 233 patients were enrolled, including 168 male patients and 65 female with a mean age of 51.4 years. One hundred and thirty-two (56.6%) patients without liver cirrhosis were designated as group 1; whereas 101(43.4%) patients with liver cirrhosis (LC) were group 2. The baseline and follow-up clinical characteristics in the two groups were shown in Table 1. There was no statistically significant difference in the demographic characteristics including age, sex, proportion of patients with diabetes mellitus (DM) and body mass index (BMI) between the two groups. Additionally, the initial and follow-up AST, ALT, HBV DNA in log scale and qHBsAg in log scale had no difference between both groups. However, there was a higher HBeAg-negative rate and high levels of total bilirubin, AFP, and initial LSM value in group 2. In addition, the patients in group 2 were treated with ETV for longer period than those in group 1 (100 weeks vs 89.4 weeks, p = 0.011). The mean duration of ETV therapy before the initial LSM was comparable between the two groups (36.6 weeks vs 38.1 weeks, p = 0.137). After ETV therapy, the log scale of HBV DNA level had a significant decrease in both groups. (3.64±2.6 IU/L to 1.26±0.6 IU/L, p<0.001 in the group 1 and 3.53±2.5 IU/L to 1.1±0.2 IU/L, p<0.001 in the group 2, respectively). Among our patients, 94 (40.3%) patients including 63 non-cirrhosis and 31 cirrhosis, were further detected their serum value of quantitative HBsAg. After ETV therapy, non-cirrhotic patients had a significant decrease of quantitative HBsAg value in log scale (3.22±0.9 IU/mL to 2.96±1.1 IU/mL, p = 0.016). However, in cirrhotic patients, there was no significant change (2.83±0.8 IU/mL to 2.8±1.1 IU/mL, p = 0.712).

**Table 1:** The demographics characteristics of patients without liver cirrhosis (Group 1) and patients with liver cirrhosis (Group 2) in the enrolment and follow-up

Characteristic	Group 1 (n = 132)	Group 2 (n = 101)	p-value
Age (mean±SD, Yr)	49.2±11.9	54.3±10.7	0.001
Male sex (%)	92(69.7)	76(75.2)	0.349
Initial AST (IU/L, mean±SD)	41.4±26.0	42.8±20.0	0.657
Initial ALT (IU/L, mean±SD)	51.2±39.3	43.4±25.3	0.083
Follow-up AST (IU/L, mean±SD)	30.7±18.6	34.7±12.1	0.058
Follow-up ALT (IU/L, mean±SD)	34.6±29.3	34.9±16.4	0.939
Initial log HBV DNA (IU/L, mean±SD)	3.64±2.6	3.53±2.5	0.478
Follow-up log HBV DNA (IU/L, mean±SD)	1.26±0.6	1.1±0.2	0.096
Initial log qHBsAg (IU/mL, mean±SD)*	3.22±0.9	2.8±1.1	0.961
Follow-up log qHBsAg (IU/mL, mean±SD)*	2.96±1.1	3.64±2.6	0.939
Total bilirubin (mg/dl, mean±SD)	0.8±0.4	1.3±0.6	<0.001
BMI (kg/m^2^, mean±SD)	24.2±3.5	24.6±3.5	0.332
AFP (ng/ml, mean±SD)	6.8±11.3	12.7±28.5	0.029
Initial LS measurement (kPa, mean±SD)	8.0±5.0	16.6±11.7	<0.001
Follow-up LS measurement (kPa, mean±SD)	6.2±3.2	13.9±10.8	<0.001
Interval of LS measurement (wks)	52.8±20.3	61.9±33.2	0.01
Therapy before initial LS measurement (wks)	36.6±45.5	38.1±49.1	0.809
Therapy before Follow-up LS measurement (wks)	89.4±49.4	100.0±57.9	0.132
Diabetes Mellitus (%)			
Yes/No	23 (17.4)/109 (82.6)	12 (11.9)/89 (88.1)	0.241
HBeAg (%)			
Positive/Negative	54 (40.9)/78 (59.1)	20 (19.8)/81 (80.2)	<0.001
Initial HBVDNA (iu/ml, %)			
<2000/≧2000	70 (53.0)/62 (47.0)	51 (50.5)/50 (49.5)	0.701
Follow-up HBV DNA (iu/ml, %)			
<2000/≧2000	131 (99.2)/1 (0.8)	101 (100)/0 (0)	0.381

Abbreviations: AST: aspartate aminotransferase; ALT: alanine aminotransferase; qHBsAg: quantitative hepatitis B virus surface antigen; BMI: body mass index AFP: alpha-fetoprotein; LS: liver stiffness; wks: weeks; HBeAg: hepatitis B virus e-antigen.

*The comparison was based on 94 patients, including 63 non-cirrhosis and 31 cirrhosis.

### The changes of transaminase level and liver stiffness value

**Figure 1:**
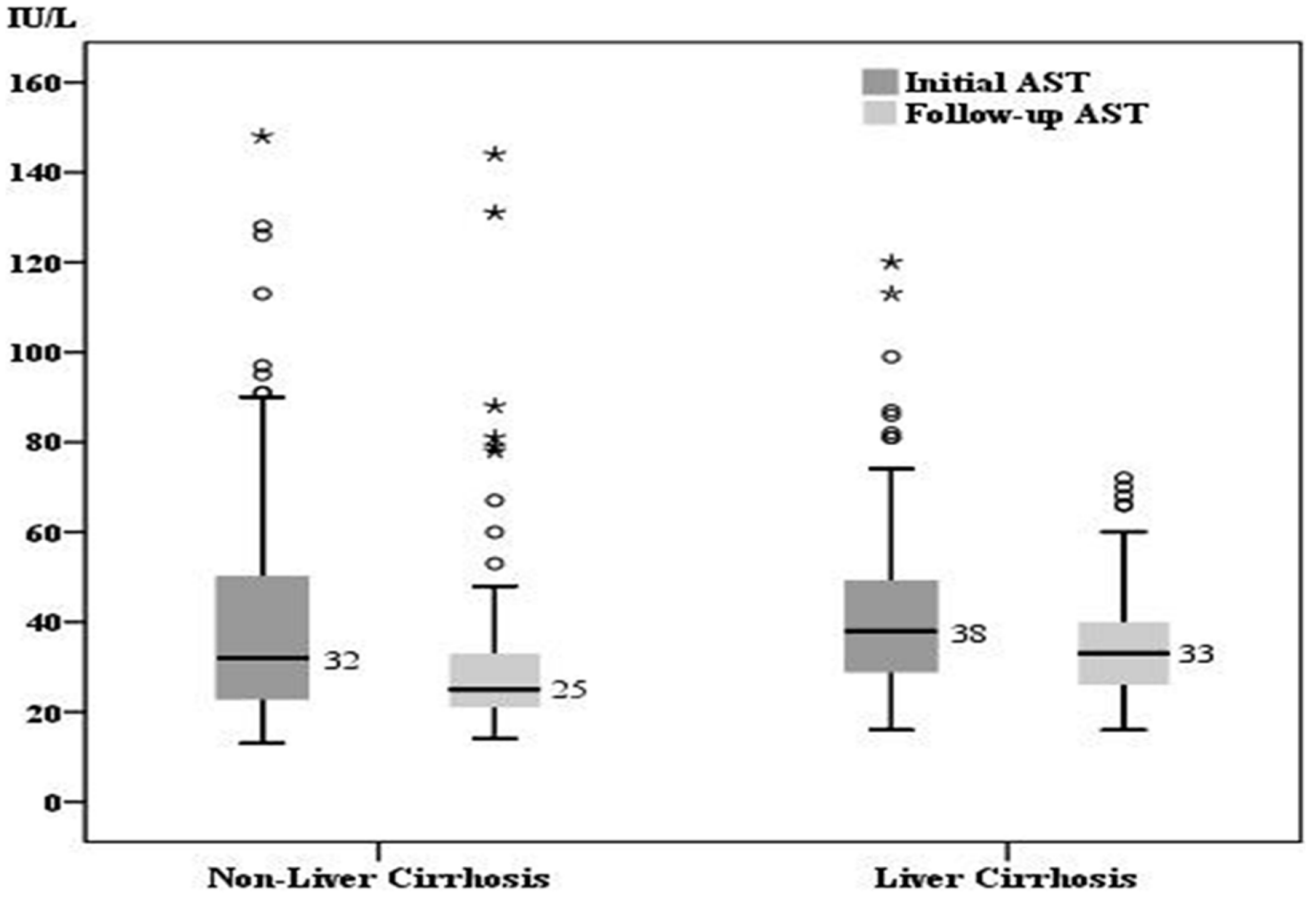
The comparison of initial and follow-up AST. For patients without liver cirrhosis (group 1) (n = 132), the median initial AST and follow-up AST was 32 IU/L and 25 IU/L, respectively. For patients with liver cirrhosis (group 2) (n = 101), the median initial AST and follow-up AST was 38 IU/L and 33 IU/L, respectively. There was a significant decrease of AST in both groups (p<0.001).

**Figure 2:**
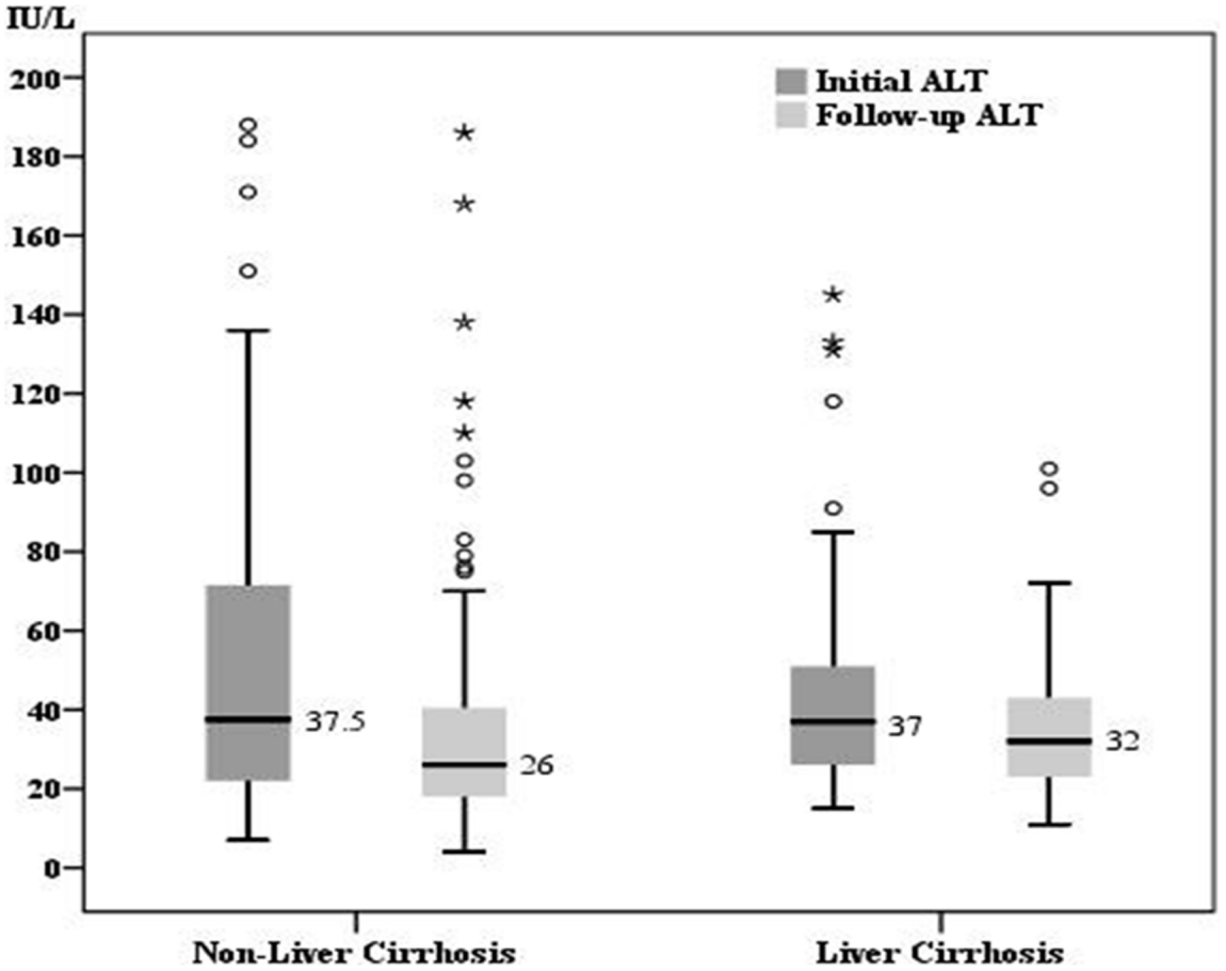
The comparison of initial and follow-up ALT. The median initial ALT and follow-up ALT was 37.5 IU/L and 26 IU/L for group 1 as well as 37 IU/L and 33 IU/L for group 2, respectively. There was a significant decrease of ALT in both groups (p<0.001).

**Figure 3:**
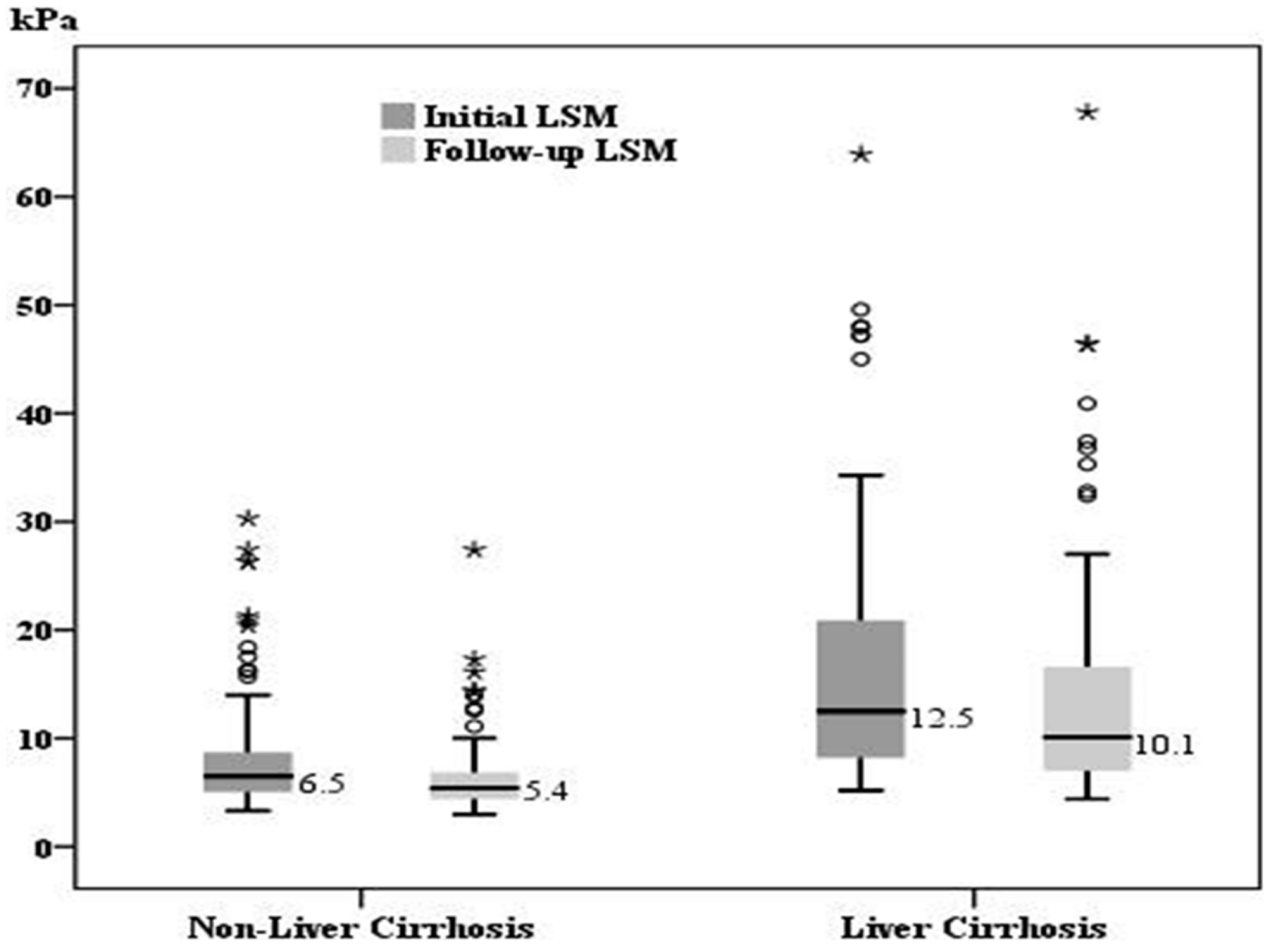
The comparison of initial and follow-up LSM. The median initial liver stiffness measurement (LSM) and follow-up LSM was 6.6 kPa and 5.4 kPa for group 1 as well as 12.4 kPa and 10.1 kPa for group 2, respectively. There was a significant decrease of LSM in both groups (p<0.001).

For the patients in group 1, the median initial and follow-up levels were 32 IU/L (range: 13–148) and 25 IU/L (range: 14–144) for AST, 37.5 IU/L (range: 7–188) and 26 IU/L(range: 4–186) for ALT, as well as 6.5 kPa (range: 3.3–30.3) and 5.4 kPa (3.0–27.4) for LSM, respectively. As for the patients in group 2, the median initial and follow-up levels were 38 IU/L(range: 16–120) and 33 IU/L(range: 16–72) for AST, 37 IU/L (range: 15–145) and 32 IU/L (range: 11–101) for ALT, as well as 12.5 kPa (range: 5.2–63.9) and 10.1 kPa (4.4–67.8) for LSM, respectively. All the data in the two groups showed a significant decline during the follow-up (p<0.001) (Figure 1–3).

### Factors associated with the changes of liver stiffness

Table 2 showed the univariate and multivariate linear regression analyses of the factors associated with the changes between two LSMs in group 1. In the univariate analysis, the factors contributing to a greater decline of LSM value included higher initial serum AFP, AST, and total bilirubin levels, lower follow-up AST, higher initial LSM value, shorter duration of ETV therapy before the initial LSM, and longer duration between the two LSMs. With regard to the multivariate analysis, lower follow-up AST level, higher initial LSM value and presence of HBeAg were associated with significant reduction of LSM value. For the patients in group 2, the univariate analysis showed that the associated factors contributing to a greater decline of LSM value included higher initial serum ASL, ALT, and AFP levels, higher initial LSM value, absence of DM and longer duration between the two LSMs (Table 3). In the multivariate analysis, longer duration between the two LSMs, higher initial LSM value and initial AST level more than 40 IU/L were associated with reduced LSM value, whereas presence of DM with increased LSM value.

**Table 2:** Linear Regression Analysis of associated factors of LS change for patients without liver cirrhosis (group 1).

	comparison	n	LS change	Univariate			Multivariate		
			(mean±SD)	Coefficients	SE	p value	Coefficients	SE	p value
Intercept							−3.407	0.355	<0.001
Sex	Female	40	2.0±5.	−0.535	0.739	0.471			
	Male	92	1.5±3.3						
Diabetes	No	109	1.7±3.8	−0.286	0.897	0.751			
	Yes	23	1.4±4.5						
Initial AST (IU/L)	≦40	84	0.9±2.3	2.112	0.683	0.002			
	>40	48	3.0±5.5						
Initial ALT (IU/L)	≦40	70	1.3±3.6	0.823	0.678	0.227			
	>40	62	2.1±4.2						
Follow-up AST (IU/L)	≦40	115	1.9±3.8	−2.050	1.001	0.043	−4.584	0.538	<0.001
	>40	17	−0.1±4.4						
Follow-up ALT (IU/L)	≦40	99	1.9±4.0	−1.012	0.781	0.197			
	>40	33	0.9±3.7						
Total bilirubin (mg/ml)	<1.4	120	1.4±3.2	2.480	1.164	0.035			
	>1.4	12	3.9±8.1						
BMI (kg/m^2^)	≦27	104	1.6±3.7	0.402	0.832	0.630			
	>27	28	2.0±4.6						
HBeAg	Negative	78	1.4±3.4	0.707	0.690	0.308	0.714	0.356	0.047
	Positive	54	2.1±4.5						
HBVDNA (iu/ml)	<2000	70	1.1±3.0	1.093	0.675	0.108			
	≧2000	62	2.2±4.7						
AFP (ng/ml)	≦15	125	1.4±3.2	4.068	1.477	0.007			
	>15	7	5.5±10.0						
Age (year)	49.2±11.9		1.7±3.9	0.028	0.029	0.322			
Interval of LS measurement (wks)	52.8±20.3			0.042	0.016	0.011			
Therapy before initial LS measurement (wks)	36.6±45.5			−0.017	0.007	0.024			
Therapy before follow-up LS measurement (wks)	89.4±49.4			−0.007	0.007	0.301			
Initial LS (kPa)	8.0±5.0			0.602	0.044	<0.001	0.675	0.036	<0.001

Abbreviations: SE: standard error; AST: aspartate aminotransferase; ALT: alanine aminotransferase; BMI: body mass index; AFP: alpha-fetoprotein; LS: liver stiffness; wks: weeks; HBeAg: hepatitis B virus e-antigen.

**Table 3:** Linear Regression Analysis of associated factors of LS change for patients with liver cirrhosis (group 2).

	comparison	n	LS change	Univariate			Multivariate		
			(mean±SD)	Coefficients	SE	p value	Coefficients	SE	p value
Intercept							−3.989	1.581	0.013
Sex	Female	25	3.9±6.3	−1.454	1.637	0.377			
	Male	76	2.4±7.3						
Diabetes	No	89	3.5±6.7	−6.302	2.099	0.003	−4.215	1.886	0.028
	Yes	12	−2.8±8.0						
Initial AST (IU/L)	≦40	59	0.4±4.9	5.801	1.316	<0.001	3.074	1.357	0.026
	>40	42	6.2±8.3						
Initial ALT (IU/L)	≦40	57	1.1±5.6	3.868	1.377	0.006			
	>40	44	5.0±8.2						
Follow-up AST (IU/L)	≦40	76	2.8±6.6	0.199	1.643	0.904			
	>40	25	2.9±8.6						
Follow-up ALT (IU/L)	≦40	73	2.5±7.0	0.915	1.582	0.564			
	>40	28	3.4±7.4						
Total bilirubin (mg/ml)	<1.4	75	2.1±6.5	2.821	1.597	0.080			
	>1.4	26	4.9±8.3						
PLT(10^3^/uL)	≧100	65	2.9±6.0	−0.406	1.480	0.784			
	<100	36	2.5±8.8						
BMI (kg/m^2^)	≦27	81	2.5±7.5	1.311	1.775	0.462			
	>27	20	3.5±5.0						
HBeAg	Negative	81	2.3±7.1	2.427	1.763	0.172			
	Positive	20	4.7±6.9						
HBVDNA (iu/ml)	<2000	51	2.2±5.1	1.259	1.413	0.375			
	≧2000	50	3.4±8.7						
AFP (ng/ml)	≦15	86	1.8±5.8	6.775	1.875	<0.001			
	>15	15	8.5±10.6						
Age (year)	54.3±10.7		2.8±7.1	−0121	0.066	0.068			
Interval of LS measurement (wks)	61.9±33.2			0.057	0.021	0.007	0.045	0.018	0.015
Therapy before initial LS measurement (wks)	38.1±49.1			−0.022	0.014	0.127			
Therapy before follow-up LS measurement (wks)	100.0±57.9			0.003	0.012	0.825			
Initial LS (kPa)	16.6±11.7			0.258	0.055	<0.001	0.193	0.057	0.013

Abbreviations: SE: standard error; AST: aspartate aminotransferase; ALT: alanine aminotransferase; PLT: platelet; BMI: body mass index; AFP: alpha-fetoprotein; LS: liver stiffness; wks: weeks; HBeAg: hepatitis B virus e-antigen.

## Discussion

In CHB patients, Osakabe et al. reported that antiviral treatment reduced LSM values at 1, 2, and 3 years after the beginning of antiviral treatment compared with the pretreatment values.[21] The reduction of LSM values by antiviral therapy was significantly correlated with the reduction of hyaluronic acid. Wong et al. also demonstrated that decrease of LSM values was related to ALT normalization during antiviral therapy.[22] It seemed that changes in LSM values are associated with the level of liver inflammation.

A previous longitudinal study reported that 1-year follow-up revealed a significant reduction in LSM value with ALT normalization in the patients with CHB and acute severe flares.[23] However, the LSM value at baseline was shown to be spuriously high due to severe acute exacerbation and not an indication of the underlying fibrosis stage. Therefore, the changes in LSM values in this condition did not reflect real reversal of fibrosis. To avoid the influence of severe inflammation, the patients with CHB receiving the initial LSM with AST or ALT levels more than 200 IU/L were excluded from the present study. Before the initial LSM was conducted, our patients have received ETV treatment with a mean duration of 36.6 weeks in group 1 and 38.1 weeks in group 2. Hence, the liver inflammation was improved with ETV treatment. Following the continued ETV treatment with a mean duration of 52.8 weeks in group 1 and 61.9 weeks in group 2, there were significant declines in AST and ALT levels in both groups. It seemed that prolonged antiviral therapy induced further normalization of AST and ALT in the patients with CHB, no matter in cirrhotic or non-cirrhotic liver.

Our previous study reported that 10 kPa was the cutoff point for diagnosis of liver cirrhosis in the patients with CHB.[25] In the present study, the patients with cirrhosis had a significant higher initial LSM value in average than those without cirrhosis (16.6 kPa vs 7.9 kPa, p<0.001). Prolonged ETV treatment contributed to a significant reduction of LSM value in both groups, indicating successful antiviral therapy might improve fibrosis over time. This is consistent with the results reporting reversal of fibrosis as determined by the histological studies after prolonged antiviral therapy.[21], [22]

The present study showed that the changes of LSM values were correlated with several biochemical and virological parameters in the patients with CHB. In the group composed of the patients without cirrhosis (group 1), those who had higher levels of initial AST, total bilirubin, and AFP achieved a greater reduction of LSM value than those had lower levels of initial AST, total bilirubin, and AFP. This indicates that patients with more severe liver inflammation at baseline could have more improvement in LSM value after receiving the same antiviral treatment. Longer duration of ETV treatment between the two LSMs contributed to a greater decline of LSM value, representing a better reversal of fibrosis. This could explain why the patients with longer duration of ETV treatment before receiving the initial LSM had a smaller decline of LSM value at the follow-up, because these patients had a relatively better liver condition at the time of enrollment. Multiple linear regression analysis demonstrated that higher initial LSM value and presence of HBeAg were associated with greater reduction of LSM value, whereas follow-up AST higher than 40 IU/L with increased LSM value. It seemed that, in non-cirrhotic CHB patients those factors associated with poorer liver parenchyma could contribute to a greater decline of LSM value at the follow-up. Similarly, in the group composed of the patients with LC (group 2), higher levels of initial AST, ALT, total bilirubin, and AFP were related to the improvement of LSM value at the follow-up. Additionally, longer duration of ETV treatment between the two LSMs seemed to associate with a better improvement of fibrosis. In the further multiple analysis, higher initial LSM value, AST level and longer duration of ETV treatment between the two measurements also induced an improvement in liver stiffness. Furthermore, we found that DM led to the progression of liver stiffness, which was not mentioned previously. Type 2 DM and HCV infection are common conditions involving;[26] indeed, comorbid DM could reduce the effect of antiviral therapy in CHC patients and resulted to a poorer outcome.[27], [28] However, less studies mentioned about the association between DM and antiviral therapy in CHB patients. Earlier studies suggested that type 2 DM was an independent factor for the development of HCC in patients with CHB, especially in those with cirrhosis.[29], [30] The present study demonstrated that DM played a role on liver stiffness progression in cirrhotic CHB patients, even under ETV treatment. This suggested that CHB patients with comorbid DM have higher risk to develop HCC and might require a closer surveillance program.

There are several limitations of the present study. Firstly, liver histology was not available in this study. Liver biopsies were not readily accepted by patients because of invasive risks. Thus, the direct association of real histological fibrosis and liver stiffness cannot be achieved due to lack of the biopsy data. Secondly, the patients who received initial LSM with AST or ALT more than 200 IU/L were excluded to avoid the influence of acute hepatitis exacerbation. As part of the enrolled patients might be at the recovery phase of hepatitis, their LSM values could not reflect the real fibrosis. This might lead to over-estimate fibrosis stage in the enrollment.

In conclusion, in addition to AST and ALT levels, LSM value was also improved in the CHB-infected patients with ETV therapy. High initial LSM value was shown to be associated with a significantly reduced of LSM value at the follow-up. However, follow-up AST more than 40 IU/L and presence of DM contributed to the increased LSM for non-cirrhotic and cirrhotic patients, respectively.
